# Delving Into PubMed Records: How AI-Influenced Vocabulary has Transformed Medical Writing since ChatGPT

**DOI:** 10.5334/pme.1929

**Published:** 2025-12-02

**Authors:** Kentaro Matsui

**Affiliations:** 1 Department of Clinical Laboratory, National Center Hospital, National Center of Neurology and Psychiatry, 4-1-1 Ogawa-Higashi, Kodaira-shi, Tokyo, 187-8551, Japan; 2 Department of Sleep-Wake Disorders, National Institute of Mental Health, National Center of Neurology and Psychiatry, 4-1-1 Ogawa-Higashi, Kodaira-shi, Tokyo, 187-8553, Japan

## Abstract

**Introduction:**

It is estimated that large language models (LLMs), including ChatGPT, are already widely used in academic paper writing. This study examined whether certain words and phrases reported as frequently used by LLMs have increased in medical literature, comparing their trends with common academic expressions.

**Methods:**

A structured literature review identified 135 potentially AI-influenced terms from 15 studies documenting LLM vocabulary patterns. For comparison, 84 common academic phrases in medical research served as controls. PubMed records from 2000 to 2024 were analyzed to track the frequency of these terms. Usage trends were normalized using a modified Z-score transformation.

**Results:**

Of the 135 potentially AI-influenced terms, 103 showed meaningful increases (modified Z-score ≥3.5) in 2024. Terms with the highest increases included “delve,” “underscore,” “primarily,” “meticulous,” and “boast.” The linear mixed-effects model revealed significantly higher usage of potentially AI-influenced terms compared to controls (β = 0.655, p < 0.001). Notably, these terms began increasing in 2020, preceding ChatGPT’s 2022 release, with marked acceleration in 2023–2024.

**Discussion:**

Certain words and phrases have become more common in medical literature since ChatGPT’s introduction. However, the use of these terms tended to increase before 2022, indicating the possibility that the emergence of LLMs amplified existing trends rather than creating entirely new patterns. By understanding which terms are overused by AI, medical educators and researchers can promote better editing of AI-assisted drafts and maintain diverse vocabulary across scientific writing.

## Introduction

ChatGPT rapidly achieved widespread global use after its launch on November 30, 2022. Trained on a vast corpus of text data, the large language model (LLM) including ChatGPT generates natural language with remarkable fluency. Shortly after its release, ChatGPT’s applicability for scientific writing in medical and biological fields became evident [1,2]. Due to the fervor surrounding its capabilities, it was credited as an author on several papers, igniting considerable debate (currently, AI is not acknowledged as an author in scholarly publications [3]). There were even opinions that the use of ChatGPT in paper writing was plagiarism [4], but in reality, LLMs such as ChatGPT, Gemini, and Claude are already being used in paper writing. The use of LLMs can be applied in various ways in academic writing [1,5] and are also important for the research activities of non-native researchers whose first language is not English [6,7,8]. A framework has been established that permits the use of LLMs in writing, provided their involvement is adequately acknowledged [3].

As the usefulness of LLMs becomes more evident, the number of researchers using them for writing papers has been gradually increasing [9,10,11,12,13,14,15,16]. Early studies on detecting text generated by LLMs have identified several frequently used words, such as ‘commendable,’ ‘meticulous,’ ‘intricate,’ and ‘realm’ [10,11,12,17]. These investigations relied on ground-truth corpora comparing human- and LLM-generated texts, but their black-box nature and sensitivity to prompt or model biases limited their generalizability. More recently, Kobak et al. analyzed over 15 million PubMed biomedical abstracts published between 2010 and 2024, examining “excess vocabulary” by extrapolating pre-ChatGPT (2021–2022) word frequencies to 2024; their analysis revealed an unprecedented post-2022 surge in stylistic words including ‘delve’ and ‘underscore’ [16]. However, no prior work has quantitatively tested whether the words and short phrases previously identified as LLM-associated have increased disproportionately compared to routine medical phrasing over the long term.

Recent discussions suggest that the lexical preferences of large language models may have been shaped by reinforcement learning from human feedback (RLHF) [18], which rewards stylistic features favored by human evaluators [19]. In this reinforcement influenced model behavior, some words that later became emblematic of LLM-generated writing might have already been increasing in usage before the public release of ChatGPT in 2022. To investigate this possibility, this study examined long-term trends in LLM-associated words and short phrases within PubMed-indexed abstracts from 2000 to 2024, testing whether their increase accelerated after the introduction of ChatGPT relative to stable control expressions.

## Materials and Methods

### Literature Search and Screening Process

To identify potentially AI-influenced terms from the literature, I conducted a structured literature review, applying a scoping review approach. I searched Scopus for both published papers and preprints, analyzing them as separate categories. I included records that either specifically reported words or phrases frequently used by LLMs compared to human writing, or focused on terms with increased usage frequency following the emergence of ChatGPT, exemplified by the “excess vocabulary” reported by Kobak et al. [16]. Studies were excluded if all identified terms had been previously reported in earlier literature. The search strategy employed the following query: TITLE-ABS-KEY ((ChatGPT OR “large language model*” OR LLM*) AND (academi* OR scientif* OR scholar* OR “peer review*”) AND (vocab* OR lexic* OR (word* W/2 (frequen* OR usage OR shift*)) OR (LLM* W/2 modif*) OR (generat* W/1 text) OR prevalen*)) AND PUBYEAR > 2021.

For data management, I used the built-in export function of Scopus to obtain published papers in CSV format. Since Scopus does not provide CSV export functionality for preprints, preprint records were extracted from the Scopus web interface and programmatically formatted to match the CSV structure of the published papers using a custom Python script (available at https://github.com/matsuikentaro1/delving_into_pubmed_records). The screening involved a two-stage approach: first, title and abstract screening against predetermined inclusion criteria, followed by a full-text review with documentation of exclusion reasons.

The search yielded 910 published records; of these, 38 passed title and abstract screening, and 11 were included after full-text review. For preprints, I identified 363 records, retained 27 after initial screening, and included 11 following full-text review. Seven records appeared in both databases. After removing duplicates, 15 unique studies [10,11,12,14,15,16,17,18,20,21,22,23,24,25,26] were included in the final analysis. A PRISMA flow diagram detailing the selection process is provided in Appendix 1.

### Identification of Potentially AI-Influenced Terms and Controls

I extracted terms from each paper that were reported as frequently used by LLMs, as well as those with a reported clear increase in usage frequency after the emergence of ChatGPT. These terms, sourced from the main text, figures, tables, and supplementary materials, were designated as “potentially AI-influenced.”Some phrases showing increased usage were also included, limited to two-word expressions. I excluded overly common words such as ‘this,’ ‘these,’ ‘through,’ and ‘their,’ as well as terms that yielded no results in PubMed searches, such as ‘while’ and ‘dive into.’ Additionally, I excluded words explicitly related to generative AI, such as ‘chatgpt,’ ‘prompt,’ and ‘diffusion,’ as they fell outside the original scope of examining characteristic word usage patterns of LLMs. Details regarding which words and phrases were extracted from each paper are provided in Appendix 2. The final list comprised 135 potentially AI-influenced terms (Table 1).

**Table 1:** Words and phrases examined for usage rates.


POTENTIALLY AI-INFLUENCED TERMS	

Verbs	

	address, align, boast, bolster, catalyze, comprehend, delve, elucidate, embark, emerge, employ, emphasize, encompass, endeavor, enhance, excel, exhibit, explore, facilitate, fortify, foster, garner, grapple, harness, highlight, illuminate, integrate, interplay, juxtapose, leverage, navigate, necessitate, offer, outperform, revolutionize, scrutinize, showcase, surpass, transform, transcend, underscore, unearth, unveil

Adjectives	

	actionable, commendable, complex, comprehensive, critical, crucial, deeper, essential, exceptional, exhaustive, expansive, fresh, fundamental, groundbreaking, ingenious, innovative, intricate, intriguing, invaluable, meticulous, multifaceted, noteworthy, nuanced, pivotal, potent, potential, renowned, significant, transformative, unlocking, valuable, versatile, well-rounded

Adverbs	

	accurately, additionally, aptly, compellingly, effectively, effortlessly, excellently, impressively, lucidly, methodically, notably, particularly, predominantly, primarily, profoundly, promptly, reportedly, scholarly, seamlessly, strategically, subsequently, thereby, thoroughly, thoughtfully, ultimately, undoubtedly

Nouns	

	advancement, capability, captive complexity, ecosystem, enhancement, essence, finding, foundation, insight, intricacy, journey, landscape, milestone, pipeline, prowess, realm, significance, soil, tapestry, testament, thought, understanding, utilization

Phrases	

	deep dive, driving force, ethical consideration, exercise caution, game changer, in addition, in summary, knowledge gap, shed light, vital role

**COMMON ACADEMIC WORDS OR PHRASES IN THE MEDICAL FIELD (CONTROL)**	

	adverse events, aim of, aimed to, all patients, analysis, associated with, association between, at least, basis of, cohort study, common grade, compared with, control group, cross-sectional, difference between, difference in, dose of, effect of, efficacy, end of, end point, evaluate, examine, further research, higher in, hypothesis, identify, included in, increased risk, independently associated, investigate, is known, less likely, logistic regression, lower in, median follow-up, models, more likely, most common, no difference, objective of, one of, our results, outcome measure, patients who, placebo group, primary endpoint, primary outcome, proportion of, purpose of, randomly, ratio, registered with, regression analysis, results suggest, retrospective, safety, secondary analysis, sought to, statistically, study period, suggest that, this study, this trial, time of, to assess, to compare, to determine, to evaluate, to examine, to identify, to investigate, to receive, treatment allocation, treatment assignment, treatment of, two groups, was found, was used, were assessed, were collected, were enrolled, were performed, were reported


For comparison, I created a control group of common academic words or short phrases (up to two words) in medical research by extracting items from the four-word lexical bundles identified in a previous study [27]. After excluding expressions that contained words listed as potentially AI-influenced terms in this study, such as ‘significant,’ ‘finding,’ and ‘potential,’ a final set of 84 PubMed-searchable expressions was obtained (Table 1).

### PubMed Search Strategy

I used PubMed’s advanced search feature (https://pubmed.ncbi.nlm.nih.gov/advanced/) to reveal the number of records in which these words were used by searching for “Text Word”. To ensure comprehensive coverage of verb forms in English, the search query included the base form, third person singular present, present participle/progressive, past tense, and past participle. For nouns, both singular and plural forms were incorporated. Considering the daily increase in records indexed in PubMed, the search conditions were standardized from January 1, 2000, to December 31, 2024. The search formulas for all words/phrases are shown in Appendix 3.

### Statistical Analysis

To investigate the usage trends of potentially AI-influenced terms in the PubMed database, I first calculated the usage frequency of each term by dividing the number of records containing the term by the total number of records in PubMed for each year from 2000 to 2024. This process yielded a dataset with usage frequency for each term and year.

Next, the modified Z-score transformation was used to normalize the usage frequency and facilitate comparisons across terms and years. For each term, the median and median absolute deviation (MAD) were calculated. The modified Z-score was computed by subtracting the median from each occurrence rate, dividing the result by the MAD, and multiplying by 0.6745. An absolute modified Z-score of 3.5 or higher was considered indicative of a meaningful increase or decrease in term usage to identify significant deviations [28], a threshold used in prior bibliometric studies [29,30]. Then, a linear mixed-effects model was used to compare the usage of potentially AI-influenced terms and common academic phrases from 2000 to 2024. The data, consisting of modified Z-scores for each word or phrase, were obtained and reshaped into a long format. The model was constructed using the ‘lme’ function from the ‘nlme’ package in R. It included the modified Z-scores as the dependent variable, the group (potentially AI-influenced terms or common academic phrases) as a fixed effect, and a random intercept for each word or phrase to account for repeated measures. The model’s summary was generated to assess the significance of the fixed effect of the group on term usage. A line plot with 95% confidence intervals was created using the ‘ggplot2’ package to visualize the trends in mean usage for each group from 2000 to 2024. In addition, as a sensitivity analysis, I repeated the model after excluding potentially AI-influenced terms with fewer than 2,000 PubMed records in 2000–2024 (bottom 10% of the distribution). The significance level for all statistical tests was set at 0.05. These analyses were performed using R version 4.3.2.

## Results

The analysis covered a total of 27,501,542 records indexed in PubMed between January 1, 2000, and December 31, 2024. The frequency rates of each word/phrase were determined using the annual total number of records as the denominator, followed by the calculation of the modified Z-score.

**Figure 1:**
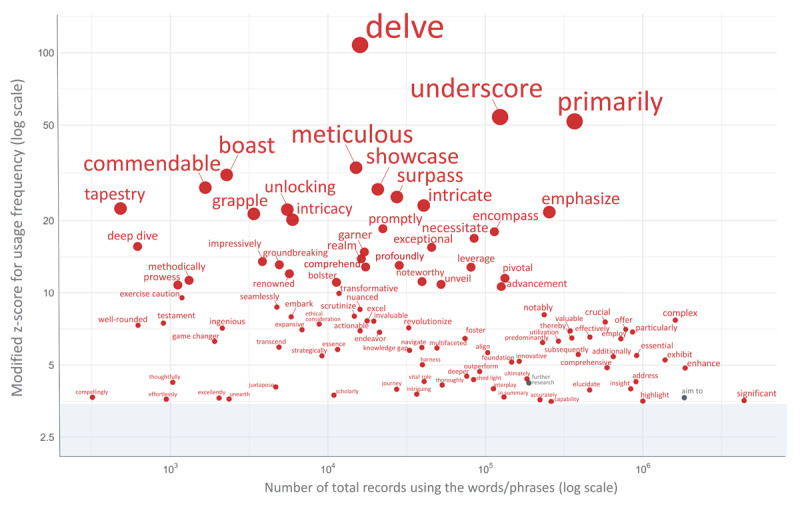
Scatter plot of word/phrase usage frequency vs. modified Z-Score in 2024. Figure 1 illustrates the relationship between the frequency of use and the modified Z-scores for words and phrases with absolute modified Z-scores exceeding 3.5 in 2024. Red circles represent potentially AI-influenced terms, while grey circles represent common academic terms in the medical field (control). The x-axis shows the number of total records using the words/phrases on a logarithmic scale, and the y-axis displays the modified Z-score for usage frequency, also on a logarithmic scale. Shaded areas represent 95% confidence intervals.

In this study, among the 135 potentially AI-influenced terms verified, 103 words/phrases exhibited a modified Z-score exceeding 3.5 in 2024. The increase was particularly pronounced for many terms at the top of this list, with ‘delve’ showing the highest score among the terms examined. Other markedly increased terms included ‘underscore,’ ‘primarily,’ ‘meticulous,’ ‘boast,’ ‘commendable,’ ‘showcase,’ ‘surpass,’ ‘intricate,’ ‘tapestry,’ and ‘unlocking’ (see Appendix 4 for the full list and Figure 1 for visual trends). While the majority of the 84 common academic phrases (controls) displayed no remarkable deviations in usage rates, ‘further research’ and ‘aim to’ also surpassed a modified Z-score of 3.5 in 2024. On the other hand, phrases such as ‘purpose of,’ ‘end of,’ ‘to determine,’ ‘hypothesis,’ ‘results suggest,’ ‘all patients,’ and ‘treatment of’ showed a notable decrease (modified Z-score < –3.5) in the same year (details in Appendix 4).

**Figure 2:**
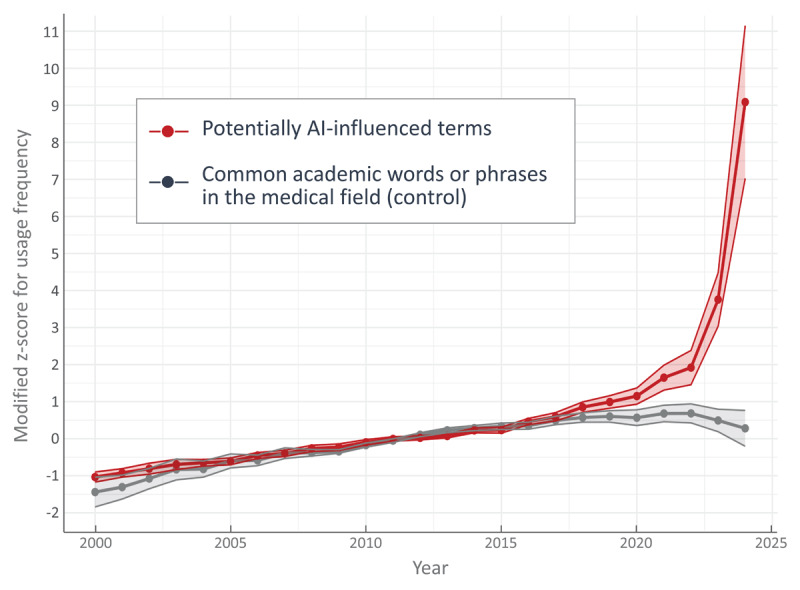
Mean usage (modified Z-scores) of potentially AI-influenced terms and common academic phrases from 2000 to 2024.

The linear mixed-effects model revealed a significant effect of the group (potentially AI-influenced terms vs. common academic phrases) on the modified Z-scores of term usage. The model showed that the usage of potentially AI-influenced terms was significantly higher than that of common academic phrases (β = 0.655, SE = 0.085, t(217) = 7.674, p < 0.001). This finding remained consistent in a sensitivity analysis excluding terms in the bottom 10% of the frequency distribution (β = 0.636, SE = 0.087, t(204) = 7.293, p < 0.001). The line plot (Figure 2) illustrates the trends in mean frequency for potentially AI-influenced terms and common academic phrases from 2000 to 2024. The frequency of the control group remained relatively stable throughout the period. In contrast, the potentially AI-influenced terms exhibited a steep upward trajectory starting in 2020, with a sharper increase observed in 2023 and 2024.

## Discussion

This study examined long-term trends in LLM-associated words and phrases in PubMed abstracts from 2000 to 2024, comparing their usage patterns with stable control expressions before and after the public release of ChatGPT. Consistent with previous findings [10,11,12,13,14,15,16,17,18,20,21,22,23,24,25,26], the findings demonstrated marked increases in potentially AI-influenced terms, with ‘delve,’ ‘underscore,’ ‘primarily,’ and ‘meticulous’ showing the most pronounced changes by 2024. Remarkably, while common academic phrases remained stable throughout the study period, these potentially AI-influenced terms began increasing in 2020, preceding ChatGPT’s public release in 2022, and showed a pronounced acceleration in 2023 to 2024.

Building upon previous studies that identified increasing trends in specific terms frequently used by LLMs, this research extended these findings through longer-term analysis, controlled comparisons (control phrases), and mixed-effects modeling, quantifying these changes as “relative increases” exceeding those of common academic expressions. The increase began in 2020 and accelerated in 2023–2024 post-release, suggesting a biphasic pattern of a “pre-trend plus post-release step-up.” The pre-release increase in specific terms could occur naturally and may already be reflected in the pretraining corpora of later LLMs, which were built from time-specific web snapshots (e.g., C4, April 2019 [31]). Such exposure could then be amplified by the RLHF during model fine-tuning, which is considered a primary cause of the increased frequency in LLMs’ use of specific expressions [18]. The acceleration in 2023–2024 likely reflects the public release, widespread adoption, and full-scale implementation of LLMs as writing assistance tools. Early adopters of AI writing assistants may have incorporated and propagated these linguistic patterns, which could have further amplified naturally occurring trends in scientific communication. Meanwhile, it has been reported that since March 2024, as AI-favored expressions became widely recognized, the use of words such as ‘delve,’ ‘intricate,’ ‘realm,’ and ‘pivotal’ has been deliberately avoided [15]. The complexity of these changes underscores the importance of continued vigilance and research to understand the evolving dynamics between AI language models and academic writing styles.

The gradual increase during 2020–2022 coincides with the period when AI-based services such as DeepL translation and Grammarly became widely adopted [32,33], potentially contributing to this trend. Although some studies suggested that machine learning–based systems can, in certain contexts, enhance lexical diversity [34,35], a larger body of evidence indicates that machine translation and grammar correction tools tend to promote simplification and lexical normalization [36,37,38,39]. However, Kobak et al. [16] reported that no clear increase in LLM-associated “excess vocabulary” was observed during the pre-ChatGPT period (e.g., 2020–2022) compared with two years earlier. This finding indicates that the influence of non-LLM translation and proofreading tools on lexical patterns was likely limited.

Interestingly, some of the common academic phrases used as controls also deviated in their proportion of use in 2024. The two phrases ‘further research’ and ‘aim to’ markedly increased in frequency of use in 2024, but since they are very commonly used expressions in medical academic writing, it would be difficult for humans to recognize that their frequency has increased. Conversely, the seven phrases ‘purpose of,’ ‘end of,’ ‘to determine,’ ‘hypothesis,’ ‘results suggest,’ ‘all patients,’ and ‘treatment of’ notably decreased in usage in 2024. When interpreting these results, we must remember that the language used in papers naturally evolves over time [40]; for example, ‘hypothesis’ and ‘results suggest’ had already been declining in frequency even before the introduction of ChatGPT. In addition, the usage rate of ‘results suggest’ showed considerable variation from year to year. However, the phrase ‘all patients’ did not show a noticeable decrease in usage frequency before 2022 but appears to have decreased substantially after 2023 (see Appendix 4). It has been reported that the usage of certain terms, such as the words ‘declare’ and ‘clearly,’ decreased after the introduction of ChatGPT [24]. Additionally, a decrease in the frequency of connectives, expressions that link sentences, such as ‘therefore,’ ‘after all,’ and ‘to begin with,’ has been noted [22]. For educators, understanding the linguistic patterns that have become less frequent in the post-LLM era may be just as critical as recognizing those promoted by LLMs, highlighting an overlooked area for future study.

It should be noted that this study does not intend to criticize the use of LLMs; for many researchers, especially non-native English speakers, these tools are increasingly valuable [6,7,8]. Rather, by identifying specific linguistic patterns—some of which, as discussed, were trending even before widespread LLM adoption—I aim to develop critical awareness. This awareness can empower medical educators and supervisors to guide authors in using LLMs more thoughtfully, refining their manuscripts to improve clarity and preserve their unique authorial voice. Educators in medical writing programs can apply these insights to encourage best practices in LLM-assisted writing; this includes guiding students and trainees to be critically aware of potentially overused terms and to actively diversify their vocabulary. Furthermore, medical educators should emphasize the importance of careful human editing to preserve the author’s unique voice and ensure originality. They should also promote an understanding among medical trainees that LLMs are best viewed as supportive tools for drafting and idea generation, rather than as final content creators.

This study is subject to several limitations. A key limitation lies in the selection process for potentially AI-influenced terms. Although I conducted a structured and comprehensive literature search using Scopus and extracted terms that had been suggested as frequently used by LLMs, the use of a single database and the fact that the screening was conducted by one researcher may have led to missed records or relevant expressions. Moreover, since the analysis was limited to terms previously reported in the literature, it is likely that there are other words or phrases favored by LLMs that were not captured in this study. Nevertheless, all potentially AI-influenced terms used here were supported by published evidence, ensuring objectivity and appropriateness for testing the study hypothesis. Secondly, temporal shifts in the frequency of word or phrase use could have been influenced by external factors such as evolving research trends and shifts in the style of scientific communication, factors not accounted for in this study. The introduction of new research topics, particularly in rapidly evolving fields such as artificial intelligence, biotechnology, and pandemic-related studies, also may have contributed to these changes in language use. Although a temporal association with the rise of LLMs was demonstrated, causal inference cannot be established; therefore, the results should be interpreted with caution. Thirdly, the application of the modified Z-score to the time series data presents limitations. The threshold for identifying significant deviations (absolute value ≥ 3.5) was based on convention rather than strict theoretical grounds. In addition, although the chosen cutoff is generally regarded as conservative [28], repeated testing across multiple terms carries a risk of false positives. While alternative approaches like time-series or Bayesian change-point models can be considered, the limited number of annual data points, especially with only two in the post-ChatGPT period (2023 and 2024), made such models unstable. This limitation stems from the nature of the PubMed dataset; consequently, future studies using more fine-grained temporal data may enable higher-resolution time-series analysis and causal estimation.

## Conclusion

This study demonstrated an increased prevalence of specific words and phrases in academic writing following the introduction of ChatGPT. The list of potentially AI-influenced terms discussed in this study can be advantageous for both users employing LLMs for writing purposes and for individuals in educational and supervisory capacities within the fields of medicine and biology. As LLMs evolve, distinguishing between human and AI-generated text may become more challenging [41]. However, simultaneously, there is a possibility that qualitative shifts in academic terminology may evolve in subtle, imperceptible ways, eluding human detection. Future research should adopt more sophisticated linguistic and bibliometric methods to track and understand these nuanced changes. In addition, qualitative approaches, such as author surveys, could help triangulate whether these linguistic patterns reflect conscious adoption of AI suggestions or natural stylistic evolution. Many researchers and medical trainees are expected to continue using LLMs for their writing—needless to say, adhering to ethical aspects and taking responsibility for the final output remain crucial aspects that medical educators must emphasize in training and mentoring when using these tools.

## Data Accessibility Statement

All data and code are available at https://github.com/matsuikentaro1/delving_into_pubmed_records.

## Additional Files

The additional files for this article can be found as follows:

10.5334/pme.1929.s1 Supplementary File 1. Appendix 1.

10.5334/pme.1929.s2 Supplementary File 2. Appendix 2.

10.5334/pme.1929.s3 Supplementary File 3. Appendix 3.

10.5334/pme.1929.s4 Supplementary File 4. Appendix 4.
