# Supplementary figures and images for: Delving Into PubMed Records: How AI-Influenced Vocabulary has Transformed Medical Writing since ChatGPT
Source: Perspect Med Educ. 2025 Dec 2;14(1):882–90. doi: 10.5334/pme.1929 (PMC12679996; doi:10.5334/pme.1929)

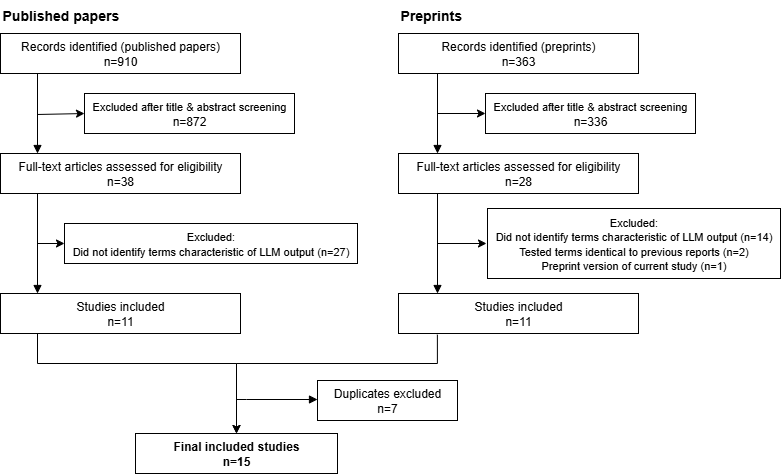

Supplement: Supplementary File 1. — Appendix 1. [file pme-14-1-1929-s1.png]
